# The In-Plane Deformation and Free Vibration Analysis of a Rotating Ring Resonator of a Gyroscope with Evenly Distributed Mass Imperfections

**DOI:** 10.3390/s25154764

**Published:** 2025-08-01

**Authors:** Dongsheng Zhang, Shuming Li

**Affiliations:** College of Aeronautical Engineering, Civil Aviation University of China, Tianjin 300300, China; 2023011035@cauc.edu.cn

**Keywords:** ring resonator, mass imperfections, steady elastic deformation, traveling waves, natural frequencies

## Abstract

A rotating imperfect ring resonator of the gyroscope is modeled by a rotating thin ring with evenly distributed point masses. The free response of the rotating ring structure at constant speed is investigated, including the steady elastic deformation and wave response. The dynamic equations are formulated by using Hamilton’s principle in the ground-fixed coordinates. The coordinate transformation is applied to facilitate the solution of the steady deformation, and the displacements and tangential tension for the deformation are calculated by the perturbation method. Employing Galerkin’s method, the governing equation of the free vibration is casted in matrix differential operator form after the separation of the real and imaginary parts with the inextensional assumption. The natural frequencies are calculated through the eigenvalue analysis, and the numerical results are obtained. The effects of the point masses on the natural frequencies of the forward and backward traveling wave curves of different orders are discussed, especially on the measurement accuracy of gyroscopes for different cases. In the ground-fixed coordinates, the frequency splitting results in a crosspoint of the natural frequencies of the forward and backward traveling waves. The finite element method is applied to demonstrate the validity and accuracy of the model.

## 1. Introduction

The frequency-modulated gyroscope is a high-precision angular rate sensor with the advantages of low power consumption, low temperature sensitivity, high bandwidth, and excellent scale factor stability. The core working element of a frequency-modulated gyroscope is the resonator, and the thin ring is one of the most commonly employed resonators in practical applications due to the simple structure and stability [1,2].

The ring-shaped structures have attracted great attention since they are widely applied in rate sensors, gears, electric motors and ultrasonic motors, etc., owing to their axial symmetry and excellent mechanical properties [3,4,5,6]. In general, the free response of the stationary ring is standing waves or the real modes, while that of the rotating ring is traveling waves or the complex modes. The resonant ring of the gyroscope works in a rotating state, and its free response is traveling waves due to the Coriolis effect. Investigations of the dynamics of the perfect rings and stationary imperfect rings are sufficient, such as research on the problems of natural frequencies [7,8,9], mode contaminations [10], wave propagations [11,12], quality factors [13], nonlinearities [14,15], and stabilities [16,17,18]. Meanwhile, studies on rotating rings with non-uniform mass or density distribution are relatively fewer.

For stationary perfect rings, except the rigid body modes, they have an elastic breathing mode, pairs of degenerate bending modes with *n*th orders (integer *n* >1), and pairs of longitudinal modes with much higher natural frequencies [7,19]. If the symmetry of the ring is broken by imperfections, the natural frequencies of the bending mode pair with certain orders can split due to the non-uniform mass or stiffness distribution [10]. For rotating perfect rings, the free response for each order is two traveling waves with branched frequencies because of the Coriolis effect [8,9]. The frequency-modulated gyroscopes measure angular rate by the frequency differences between two traveling waves. The nonlinear phenomenon can be aroused by the electrostatic force for the rotating ring subjected to a non-uniform electric field [17], or the geometric nonlinearity is considered [15]. Also, parametric instabilities can result from the time-varying rotating speed [16] or structure eccentricity [20].

For rotating rings with imperfections, Kim et al. [21] analyzed the thermoelastic effect on the rotating ring with non-uniform density under the inextensional assumption, and the frequency expression included the variable density. The longitude deformation along the rotating ring is ignored in most research since it is really small, especially for low-speed conditions [22,23]. Nevertheless, it is better to consider the transverse deformation due to imperfections because it is on a larger scale than the longitudinal one and leads to variation in the normal stress, which can even cause parametrical instability for varying speed conditions.

The mathematic model of the rotating ring can be established in ground-fixed coordinates or body-fixed coordinates. By using Hamilton’s principle, the effect of circumferential stress on strain energy is directly involved if the energies are calculated in the ground-fixed coordinates [11,12], whereas the strain energy caused by the centrifugal force needs to be considered separately if it is modeled in the body-fixed coordinates [9]. A similar problem is explored by Zhang et al. [24] in the ring-fixed coordinate, and the frequency splitting phenomenon cannot be observed by setting the speed to zero in the model of the rotating ring.

From a practical point of view, it is difficult to eliminate factors such as machining errors and uneven materials in a small mechanical part, and mass imperfections on the ring resonator of the gyroscope are inevitable [13,21] and, thus, influence the vibration characteristics of the ring resonator. This work considers the ring resonator of the frequency-modulated gyroscope where the mass imperfections are evenly distributed, aiming to provide a more accurate analysis for the rotating thin ring structure with equally spaced mass imperfections. The results can lay the foundation for further research, for instance, on the effects of variable rotating speed, the nonlinearities from the large deformation, and the free response of the rotating thin ring structure with random mass imperfections. The numerical results of the natural frequencies are compared with those obtained from the finite element method, demonstrating the validity and accuracy of the present work.

## 2. Dynamic Modeling

### 2.1. Model Description

**Figure 1:**
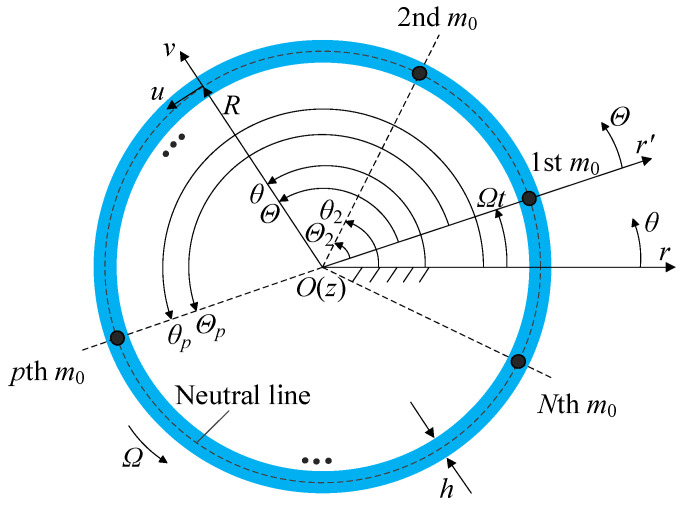
Schematic of a rotating ring structure with *N* evenly distributed point masses.

Figure 1 presents the rotating ring resonator model of the gyroscope with mass imperfections, where *O-rθz* is the ground-fixed coordinates and *O-r*′*Θz* is the ring-fixed coordinates. The origin *O* is located at the geometric center, and the *z*-axis is pointing out of the paper. The polar axes of the two coordinates coincide at the initial moment. To facilitate further investigation into the free response of the rotating ring resonator with random mass imperfections, this study analyzes the case in which the mass imperfections are evenly distributed. The mass imperfections are represented by point masses in the dynamic model. *N* is the number of the point masses, *m*_0_ is the point mass, *p* is the ordinal number of the point masses, *R* is the neutral circle radius, *b* is the axial height, *h* is the radial thickness that is much smaller than the neutral circle radius and satisfies the thin ring assumption, and the extensional deformation of the model can be neglected for micro-bending vibration. *ρ*, *E*, and *Ω* are the material density, Young’s modulus, and rotational speed, respectively.

The *u*(*θ*, *t*) and *v*(*θ*, *t*) are the tangential and radial displacements of any point on the neutral circle in the ground-fixed coordinates, respectively, which are functions of angle *θ* and time *t*.

### 2.2. Equations of Motion

The dynamic model is established in the ground-fixed coordinates, and the *Dirac* function is employed to represent the linear density of the point masses:

(1) $$m_{\theta }\left(\theta ,t\right)=m_{0}\sum_{p=1}^{N}\delta \left[\theta -\left(\Theta_{p}+\Omega t-2k\pi \right)\right]$$

where *δ* is the *Dirac* function and *k* is an integer to ensure that

$$0\leq \left(\Theta_{p}+\Omega t-2k\pi \right)\leq 2\pi$$

The ring is treated as the Euler–Bernoulli beam, and the energy method is to be utilized. The displacement vector of any point (*R*, *θ*) on the neutral circle is

(2) $$r=\left(R+v\right)e_{r}+ue_{\theta }$$

where **e***_r_* and **e***_θ_* are the radial and tangential unit vectors.

The velocity is determined by the time derivative of the position vector:

(3) $$\dot{r}=\left[\frac{𝜕v}{𝜕t}+\left(\frac{𝜕v}{𝜕\theta }-u\right)\Omega \right]e_{r}+\left[\frac{𝜕u}{𝜕t}+\left(R+v+\frac{𝜕u}{𝜕\theta }\right)\Omega \right]e_{\theta }$$

The cross-section taken normal to the middle surface of the ring remains normal after deformation. Neglecting the rotary inertia effects, the kinetic energy can be expressed as

(4) $$T=\frac{1}{2}\int_{0}^{2\pi }\left(\rho AR+m_{\theta }\right)\left\{{\left[\frac{𝜕v}{𝜕t}+\Omega \left(\frac{𝜕v}{𝜕\theta }-u\right)\right]}^{2}+{\left[\frac{𝜕u}{𝜕t}+\left(R+v+\frac{𝜕u}{𝜕\theta }\right)\Omega \right]}^{2}\right\}d\theta$$

where *A* (*A* = *bh*) is the cross-sectional area.

The tangential strain anywhere on the cross-section of the ring [12] is

(5) $$\varepsilon_{\theta }=\frac{1}{R}\left(\frac{𝜕u}{𝜕\theta }+v\right)+\frac{1}{2R^{2}}{\left(u-\frac{𝜕v}{𝜕\theta }\right)}^{2}+\frac{r-R}{R^{2}}\left(\frac{𝜕u}{𝜕\theta }-\frac{{𝜕}^{2}v}{𝜕\theta^{2}}\right)$$

According to Equation (5), the tangential strain of an arbitrary point on the neutral circle is given by

(6) $$\varepsilon_{\theta 0}=\frac{1}{R}\left(\frac{𝜕u}{𝜕\theta }+v\right)+\frac{1}{2R^{2}}{\left(u-\frac{𝜕v}{𝜕\theta }\right)}^{2}$$

In the ground-fixed coordinates, the normal stress of the cross-section is not needed to be calculated separately for the potential energy caused by the centrifugal tension [12], unlike the analysis in Ref. [9]. The constitutive equation used in this work is Hooke’s Law, that is, the stress *σ* equals to *Eε_θ_*. Then, the strain energy is

(7) $$U=\frac{1}{2}\int_{0}^{2\pi }\int_{A}E\varepsilon_{\theta }^{2}RdAd\theta$$

Assuming that the vibration amplitude and the deformation are minimal, the high terms in the strain energy are neglected. Substitution of the strain in Equation (5) into the strain energy and simplification gives

(8) $$U=\frac{1}{2}\frac{EA}{R}\int_{0}^{2\pi }\left[{\left(\frac{𝜕u}{𝜕\theta }+v\right)}^{2}+\frac{h^{2}}{12R^{2}}{\left(\frac{𝜕u}{𝜕\theta }-\frac{{𝜕}^{2}v}{𝜕\theta^{2}}\right)}^{2}+\frac{1}{R}\left(\frac{𝜕u}{𝜕\theta }+v\right){\left(u-\frac{𝜕v}{𝜕\theta }\right)}^{2}\right]d\theta$$

The Hamilton’s principle is employed:

(9) $$\delta \int_{t_{1}}^{t_{2}}(T-U)dt=0$$

Substituting Equations (4) and (8) into Equation (9) yields the partial differential equations for *v* and *u*:

(10) $$\begin{array}{l} \frac{𝜕m_{\theta }}{𝜕\theta }\Omega \frac{𝜕v}{𝜕t}+\frac{𝜕m_{\theta }}{𝜕\theta }\Omega^{2}\left(\frac{𝜕v}{𝜕\theta }-u\right)+2\left(\rho AR+m_{\theta }\right)\Omega \left(\frac{{𝜕}^{2}v}{𝜕\theta 𝜕t}-\frac{𝜕u}{𝜕t}\right)+\frac{EA}{2R^{2}}{\left(u-\frac{𝜕v}{𝜕\theta }\right)}^{2}\\ -\left(\rho AR+m_{\theta }\right)\Omega^{2}\left(v+2\frac{𝜕u}{𝜕\theta }-\frac{{𝜕}^{2}v}{𝜕\theta^{2}}\right)+m_{0}\frac{𝜕v}{𝜕t}\sum_{p=1}^{N}\frac{𝜕\delta \left(\theta -\theta_{p}\right)}{𝜕t}+\frac{EA}{R}\left(\frac{𝜕u}{𝜕\theta }+v\right)\\ +m_{0}\Omega \left(\frac{𝜕v}{𝜕\theta }-u\right)\sum_{p=1}^{N}\frac{𝜕\delta \left(\theta -\theta_{p}\right)}{𝜕t}+\left(\rho AR+m_{\theta }\right)\frac{{𝜕}^{2}v}{𝜕t^{2}}-\frac{EAh^{2}}{12R^{3}}\left(\frac{{𝜕}^{3}u}{𝜕\theta^{3}}-\frac{{𝜕}^{4}v}{𝜕\theta^{4}}\right)\\ +\frac{EA}{R^{2}}\left[\left(\frac{{𝜕}^{2}u}{𝜕\theta^{2}}+\frac{𝜕v}{𝜕\theta }\right)\left(u-\frac{𝜕v}{𝜕\theta }\right)+\left(\frac{𝜕u}{𝜕\theta }+v\right)\left(\frac{𝜕u}{𝜕\theta }-\frac{{𝜕}^{2}v}{𝜕\theta^{2}}\right)\right]=\left(\rho AR+m_{\theta }\right)\Omega^{2}R \end{array}$$

(11) $$\begin{array}{l} \frac{𝜕m_{\theta }}{𝜕\theta }\Omega \left[\frac{𝜕u}{𝜕t}+\left(v+\frac{𝜕u}{𝜕\theta }\right)\Omega \right]+2\left(\rho AR+m_{\theta }\right)\Omega \left(\frac{{𝜕}^{2}u}{𝜕\theta 𝜕t}+\frac{𝜕v}{𝜕t}\right)+\left(\rho AR+m_{\theta }\right)\frac{{𝜕}^{2}u}{𝜕t^{2}}\\ +m_{0}\frac{𝜕u}{𝜕t}\sum_{p=1}^{N}\frac{𝜕\delta \left(\theta -\theta_{p}\right)}{𝜕t}+m_{0}\Omega \left(v+\frac{𝜕u}{𝜕\theta }\right)\sum_{p=1}^{N}\frac{𝜕\delta \left(\theta -\theta_{p}\right)}{𝜕t}+\frac{𝜕m_{\theta }}{𝜕\theta }\Omega^{2}R\\ +\left(\rho AR+m_{\theta }\right)\Omega^{2}\left(2\frac{𝜕v}{𝜕\theta }+\frac{{𝜕}^{2}u}{𝜕\theta^{2}}-u\right)-\frac{EAh^{2}}{12R^{3}}\left(\frac{{𝜕}^{2}u}{𝜕\theta^{2}}-\frac{{𝜕}^{3}v}{𝜕\theta^{3}}\right)+\frac{EA}{R^{2}}\left(\frac{𝜕u}{𝜕\theta }+v\right)\left(u-\frac{𝜕v}{𝜕\theta }\right)\\ -\frac{EA}{R}\left(\frac{{𝜕}^{2}u}{𝜕\theta^{2}}+\frac{𝜕v}{𝜕\theta }\right)-\frac{EA}{2R^{2}}\frac{𝜕\left[{\left(u-𝜕v/𝜕\theta \right)}^{2}\right]}{𝜕\theta }=-m_{0}\Omega R\sum_{p=1}^{N}\frac{𝜕\delta \left(\theta -\theta_{p}\right)}{𝜕t} \end{array}$$

where *θ_p_* = 2*π*(*p* − 1)/*N* + *Ωt*.

## 3. Model Analysis

### 3.1. Solution Strategy

In the ground-fixed coordinates, the partial differential dynamical equations of the model contain time-varying coefficients, making them challenging to solve. For the purpose of the calculation of the steady elastic deformation and nature frequencies, the model is converted into the ring-fixed coordinates through coordinate transformation *θ* =*Θ* + *Ωt*. The *µ*(*Θ*, *t*) and *υ*(*Θ*, *t*) are used to represent the corresponding tangential and radial displacements in the ring-fixed coordinates. The partial differential dynamical equations for *µ* and *υ* are

(12) $$\begin{array}{l} \left[\rho AR+m_{0}\sum_{p=1}^{N}\delta \left(\Theta -\Theta_{p}\right)\right]\left(\frac{{𝜕}^{2}\upsilon }{𝜕t^{2}}-2\frac{𝜕\mu }{𝜕t}\Omega -\upsilon \Omega^{2}\right)-\frac{EAh^{2}}{12R^{3}}\left(\frac{{𝜕}^{3}\mu }{𝜕\Theta^{3}}-\frac{{𝜕}^{4}\upsilon }{𝜕\Theta^{4}}\right)\\ +\frac{EA}{R^{2}}\left(\frac{{𝜕}^{2}\mu }{𝜕\Theta^{2}}+\frac{𝜕\upsilon }{𝜕\Theta }\right)\left(\mu -\frac{𝜕\upsilon }{𝜕\Theta }\right)+\frac{EA}{R^{2}}\left(\frac{𝜕\mu }{𝜕\Theta }+\upsilon \right)\left(\frac{𝜕\mu }{𝜕\Theta }-\frac{{𝜕}^{2}\upsilon }{𝜕\Theta^{2}}\right)\\ +\frac{EA}{R}\left(\frac{𝜕\mu }{𝜕\Theta }+\upsilon \right)+\frac{EA}{2R^{2}}{\left(\mu -\frac{𝜕\upsilon }{𝜕\Theta }\right)}^{2}=\Omega^{2}R\left[\rho AR+m_{0}\sum_{p=1}^{N}\delta \left(\Theta -\Theta_{p}\right)\right] \end{array}$$

(13) $$\begin{array}{l} \left[\rho AR+m_{0}\sum_{p=1}^{N}\delta \left(\Theta -\Theta_{p}\right)\right]\left(\frac{{𝜕}^{2}\mu }{𝜕t^{2}}+2\frac{𝜕\upsilon }{𝜕t}\Omega -\mu \Omega^{2}\right)-\frac{EA}{R^{2}}\left(\mu -\frac{𝜕\upsilon }{𝜕\Theta }\right)\left(\frac{𝜕\mu }{𝜕\Theta }-\frac{{𝜕}^{2}\upsilon }{𝜕\Theta^{2}}\right)\\ -\frac{EA}{R}\left(\frac{{𝜕}^{2}\mu }{𝜕\Theta^{2}}+\frac{𝜕\upsilon }{𝜕\Theta }\right)-\frac{EAh^{2}}{12R^{3}}\left(\frac{{𝜕}^{2}\mu }{𝜕\Theta^{2}}-\frac{{𝜕}^{3}\upsilon }{𝜕\Theta^{3}}\right)+\frac{EA}{R^{2}}\left(\frac{𝜕\mu }{𝜕\Theta }+\upsilon \right)\left(\mu -\frac{𝜕\upsilon }{𝜕\Theta }\right)=0 \end{array}$$

where *Θ_p_* = 2*π*(*p* − 1)/*N*.

The partial differential dynamic equations for *µ* and *υ* are time-invariant coefficients, and the characteristics of the modes can be solved through the eigenvalue analysis.

The ring resonator in a gyroscope is assumed to be extensible with the centrifugal effect at the rotational speed *Ω* and to be inextensible during the micro-bending vibration based on the steady deformation. The radial and tangential displacements on the neutral circle can be expressed as

(14) $$\upsilon_{final}\left(\Theta ,\;t\right)=\upsilon_{e}\left(\Theta \right)+\upsilon \left(\Theta ,\;t\right)$$

(15) $$\mu_{final}\left(\Theta ,\;t\right)=\mu_{e}\left(\Theta \right)+\mu \left(\Theta ,\;t\right)$$

where *υ_final_* and *µ_final_* are the total radial and tangential displacements, *υ_e_* and *µ_e_* are the radial and tangential displacements of the steady elastic deformation, and *υ* and *µ* are the radial and tangential displacements for the inextensible vibration.

### 3.2. Steady Elastic Deformation

Due to the influence of the mass imperfections, the shape of the steady elastic deformation is not circular but a rotationally symmetric shape with an angular period related to the number of the mass imperfections *N*. The steady elastic deformation of the model can be approximated as the superposition of the steady elastic deformation of a perfect rotating thin ring and the deformation induced by the influence of the mass imperfections. The radial and tangential displacements on the neutral circle are independent of time *t* and depend on the angle *Θ*. Correspondingly, the radial and tangential displacements on the neutral circle are expressed as perturbation forms:

(16) $$\upsilon_{e}\left(\Theta \right)=\upsilon_{e0}\left(\Theta \right)+\varepsilon_{m0}\upsilon_{e1}\left(\Theta \right)$$

(17) $$\mu_{e}\left(\Theta \right)=\mu_{e0}\left(\Theta \right)+\varepsilon_{m0}\mu_{e1}\left(\Theta \right)$$

where *υ_e_*_0_ and *µ_e_*_0_ are the radial and tangential displacements of the perfect ring and *υ_e_*_1_ and *µ_e_*_1_ are the additional radial and tangential displacements induced by the influence of the mass imperfections. *ε_m_*_0_ is a small parameter, and *ε_m_*_0_ = *m*_0_/*ρAR*.

Substituting Equations (14) and (15) into Equations (12) and (13), for moderate speeds and stiff rings, the extensional deformation during rotation is small, and the nonlinear terms in the equations can be neglected. The partial differential equations for *υ_e_* and *µ_e_* are

(18) $$\frac{EAh^{2}}{12R^{3}}\left(\frac{{𝜕}^{4}\upsilon_{e}}{𝜕\Theta^{4}}-\frac{{𝜕}^{3}\mu_{e}}{𝜕\Theta^{3}}\right)+\frac{EA}{R}\left(\frac{𝜕\mu_{e}}{𝜕\Theta }+\upsilon_{e}\right)=R\Omega^{2}\left[\rho A\left(R+\upsilon_{e}\right)+\frac{\upsilon_{e}+R}{R}m_{0}\sum_{p=1}^{N}\delta \left(\Theta -\Theta_{p}\right)\right]$$

(19) $$-\rho AR\Omega^{2}\mu_{e}-m_{0}\Omega^{2}\mu_{e}\sum_{p=1}^{N}\delta \left(\Theta -\Theta_{p}\right)-\frac{EA}{R}\left(\frac{{𝜕}^{2}\mu_{e}}{𝜕\Theta^{2}}+\frac{𝜕\upsilon_{e}}{𝜕\Theta }\right)-\frac{EAh^{2}}{12R^{3}}\left(\frac{{𝜕}^{2}\mu_{e}}{𝜕\Theta^{2}}-\frac{{𝜕}^{3}\upsilon_{e}}{𝜕\Theta^{3}}\right)=0$$

Substituting Equations (16) and (17) into Equations (18) and (19) and separating the equations by orders of *ε_m_*_0_ yield

$\varepsilon_{m0}^{0}$:

(20) $$-\rho AR\Omega^{2}\upsilon_{e0}-\frac{EAh^{2}}{12R^{3}}\left(\frac{{𝜕}^{3}\mu_{e0}}{𝜕\Theta^{3}}-\frac{{𝜕}^{4}\upsilon_{e0}}{𝜕\Theta^{4}}\right)+\frac{EA}{R}\left(\frac{𝜕\mu_{e0}}{𝜕\Theta }+\upsilon_{e0}\right)=\rho AR^{2}\Omega^{2}$$

(21) $$-\rho AR\Omega^{2}\mu_{e0}-\frac{EA}{R}\left(\frac{{𝜕}^{2}\mu_{e0}}{𝜕\Theta^{2}}+\frac{𝜕\upsilon_{e0}}{𝜕\Theta }\right)-\frac{EAh^{2}}{12R^{3}}\left(\frac{{𝜕}^{2}\mu_{e0}}{𝜕\Theta^{2}}-\frac{{𝜕}^{3}\upsilon_{e0}}{𝜕\Theta^{3}}\right)=0$$

$\varepsilon_{m0}^{1}$:

(22) $$\begin{array}{l} -\varepsilon_{m0}\rho AR\Omega^{2}\upsilon_{e1}-\varepsilon_{m0}\rho AR\Omega^{2}\upsilon_{e0}\sum_{p=1}^{N}\delta \left(\Theta -\Theta_{p}\right)-\varepsilon_{m0}\frac{EAh^{2}}{12R^{3}}\left(\frac{{𝜕}^{3}\mu_{e1}}{𝜕\Theta^{3}}-\frac{{𝜕}^{4}\upsilon_{e1}}{𝜕\Theta^{4}}\right)\\ +\varepsilon_{m0}\frac{EA}{R}\left(\frac{𝜕\mu_{e1}}{𝜕\Theta }+\upsilon_{e1}\right)=\varepsilon_{m0}\rho AR^{2}\Omega^{2}\sum_{p=1}^{N}\delta \left(\Theta -\Theta_{p}\right) \end{array}$$

(23) $$\begin{array}{l} -\varepsilon_{m0}\rho AR\Omega^{2}\mu_{e1}-\varepsilon_{m0}\rho AR\Omega^{2}\mu_{e0}\sum_{p=1}^{N}\delta \left(\Theta -\Theta_{p}\right)-\varepsilon_{m0}\frac{EA}{R}\left(\frac{{𝜕}^{2}\mu_{e1}}{𝜕\Theta^{2}}+\frac{𝜕\upsilon_{e1}}{𝜕\Theta }\right)\\ -\varepsilon_{m0}\frac{EAh^{2}}{12R^{3}}\left(\frac{{𝜕}^{2}\mu_{e1}}{𝜕\Theta^{2}}-\frac{{𝜕}^{3}\upsilon_{e1}}{𝜕\Theta^{3}}\right)=0 \end{array}$$

Due to the periodicity of the radial and tangential displacements with respect to the angle *Θ*, they are expressed in the form of a Fourier series as

(24) $$\upsilon =a_{\upsilon }+\sum_{j=1}^{\infty }b_{\upsilon j}\cos j\Theta +\sum_{j=1}^{\infty }c_{\upsilon j}\sin j\Theta$$

(25) $$\mu =a_{\mu }+\sum_{j=1}^{\infty }b_{\mu j}\cos j\Theta +\sum_{j=1}^{\infty }c_{\mu j}\sin j\Theta$$

Substituting Equations (24) and (25) into Equations (22) and (23), there are

(26) $$\left\{\begin{array}{l} \upsilon_{e0}=\frac{\rho R^{3}\Omega^{2}}{E-\rho R^{2}\Omega^{2}}\\ \mu_{e0}=0 \end{array}\right.$$

(27) $$\left\{\begin{array}{l} \upsilon_{e1}=\frac{NR\alpha }{\pi \left(\alpha -1\right)}\sum_{j=N,2N\ldots }^{\infty }\left[\frac{\left(\beta +\alpha \right)j^{2}-1}{\varphi \left(j\right)}\cos j\Theta \right]\\ \mu_{e1}=\frac{NR\alpha }{\pi \left(\alpha -1\right)}\sum_{j=N,2N\ldots }^{\infty }\left[\frac{-\left(\beta j^{3}+\alpha j\right)}{\varphi \left(j\right)}\sin j\Theta \right] \end{array}\right.$$

According to Equations (16), (17), (26) and (27), the radial and tangential displacements on the neutral circle are

(28) $$\left\{\begin{array}{l} \upsilon_{e}=\frac{\rho AR^{3}\Omega^{2}}{EA-\rho AR^{2}\Omega^{2}}+\frac{\varepsilon_{m_{0}}NR\alpha }{\pi \left(\alpha -1\right)}\sum_{j=N,\;2N\ldots }^{\infty }\left[\frac{\left(\beta +\alpha \right)j^{2}-1}{\varphi \left(j\right)}\cos j\Theta \right]\\ \mu_{e}=\frac{\varepsilon_{m_{0}}NR\alpha }{\pi \left(\alpha -1\right)}\sum_{j=N,\;2N\ldots }^{\infty }\left[\frac{-\left(\beta j^{3}+\alpha j\right)}{\varphi \left(j\right)}\sin j\Theta \right] \end{array}\right.$$

where *α* = *E/ρR*^2^*Ω*^2^, *β* = *Eh*^2^*/*12*ρR*^4^*Ω*^2^, and *φ*(*j*) = *αβj*^6^ − (2*αβ* + *β*)*j*^4^ − (*α* + *β* − *αβ*)*j*^2^ + 1 − *α*.

Table 1 shows the basic parameters of the model. The small parameter *ε_m_*_0_ is calculated to be approximately 0.1343.

**Figure 2:**
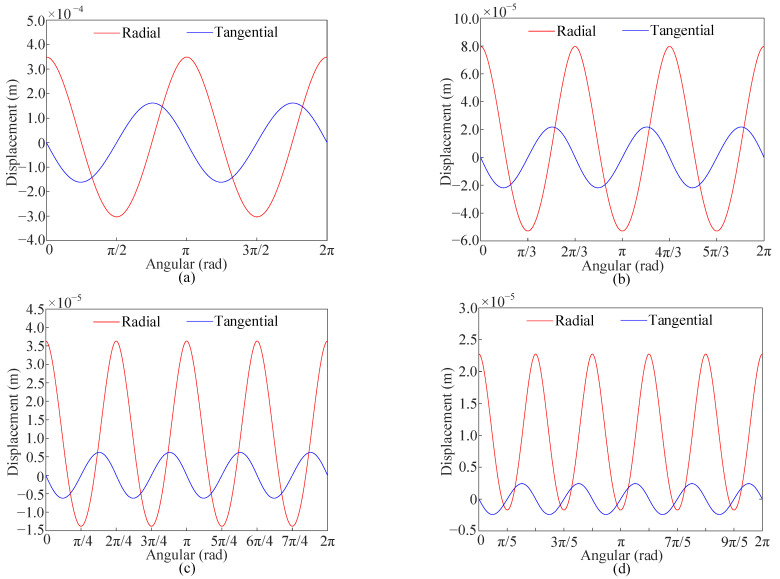
The radial and tangential displacement distribution on the neutral circle for steady elastic deformation for (**a**) *N* = 2, (**b**) *N* = 3, (**c**) *N* = 4, and (**d**) *N* = 5.

**Figure 3:**
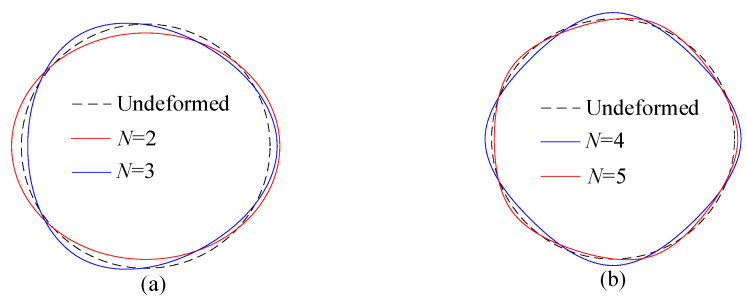
Steady elastic deformation of the model with point masses for (**a**) *N* = 2 and *N* = 3, and (**b**) *N* = 4 and *N* = 5.

Figure 2 exhibits the displacements of the points on the neutral circle for steady elastic deformation. The red and blue lines indicate the radial and tangential displacements, respectively. Figure 3 shows the steady elastic deformation diagrams obtained from Equation (28).

It is shown that the radial and tangential displacements are periodic with respect to the angle *Θ*, and the angular period is 2*π*/*N*. The amplitudes of both the radial and tangential displacements decrease as the number of the mass imperfections increases, and the radial displacement gradually becomes the dominant component of the steady elastic deformation.

The maximum radial displacement occurs at the locations of the mass imperfections (*Θ* = 2*π*(*p* − 1)/*N*), while the minimum radial displacement occurs at the midpoints between two adjacent mass imperfections (*Θ* = *π*(2*p* − 1)/*N*). At both locations, the tangential displacement is zero. The maximum tangential displacement occurs at angles *πp*/2*N* and 3*πp*/2*N*, where the magnitudes are equal but the directions are opposite. At the maximum tangential displacement, the radial displacement equals *υ_e_*_0_, which corresponds to the radial displacement of a perfect rotating ring. This indicates that, at the maximum tangential displacement, the influence of the mass imperfections on the radial displacement is negligible.

According to Equation (6), for moderate speeds and stiff rings, the extensional deformation during rotation is small, and the quadratic term can be neglected. The tangential strain on the neutral circle is

(29) $$\varepsilon_{\Theta 0}=\frac{1}{R}\left(\frac{𝜕\mu_{e}}{𝜕\Theta }+\upsilon_{e}\right)=\frac{\upsilon_{e0}}{R}+\frac{\varepsilon_{m_{0}}N\alpha }{\pi \left(\alpha -1\right)}\sum_{j=N,\;2N\ldots }^{\infty }\left[\frac{-\beta j^{4}+\beta j^{2}-1}{\varphi \left(j\right)}\cos j\Theta \right]$$

Then, the tangential tension on the neutral circle is

(30) $$N_{\Theta }=\frac{EA}{R}\left(\frac{𝜕\mu_{e}}{𝜕\Theta }+\upsilon_{e}\right)=N_{\Theta 1}+N_{\Theta 2}$$

The tangential tension is divided into two components, the tangential tension *N_Θ_*_1_ of the corresponding perfect rotating thin ring and the additional tension *N_Θ_*_2_ affected by the mass imperfections:

$$N_{\Theta 1}=\frac{EA\upsilon_{e0}}{R},\;N_{\Theta 2}=\frac{\varepsilon_{m_{0}}EAN\alpha }{\pi \left(\alpha -1\right)}\sum_{j=N,\;2N\ldots }^{\infty }\left[\frac{-\beta j^{4}+\beta j^{2}-1}{\varphi \left(j\right)}\cos j\Theta \right]$$

**Figure 4:**
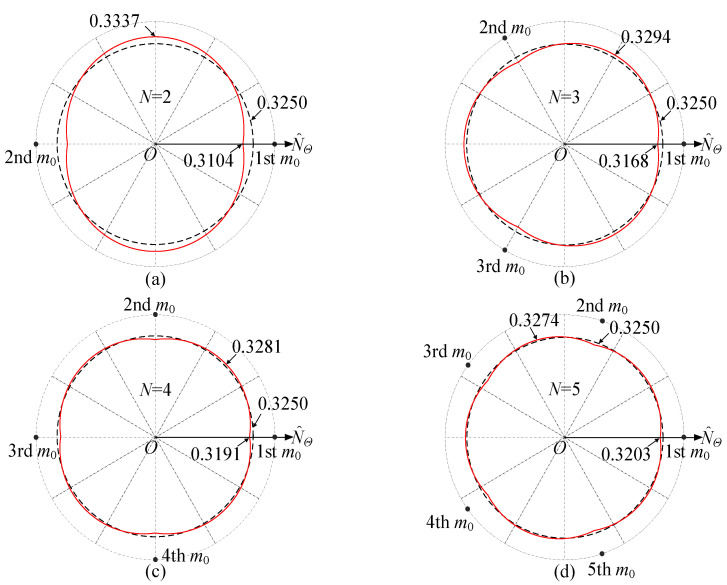
Nondimensional tangential tension distribution with different numbers of mass imperfections when *Ω* = 500 rad/s, where the straight curves ‘—’ denote the results for the perfect ring and the dashed curves ‘—’ denote the cases of (**a**) *N* = 2, (**b**) *N* = 3, (**c**) *N* = 4, and (**d**) *N* = 5.

The strain follows the same trend as the tangential tension, and it is sufficient to analyze one of them. Figure 4 provides the nondimensional tangential tension on the neutral circle, with different numbers of the mass imperfections based on the parameters in Table 1, where ${\hat{N}}_{\Theta }$ = *N_Θ_R*^2^/*EI* and *I* = *Ah*^2^/12. It can be observed that the presence of the evenly distributed mass imperfections leads to a redistribution of tangential tension in the rotating ring by comparing the tangential tension distribution without the mass imperfections. Specifically, the angular period of the tangential tension distribution with evenly distributed mass imperfections is 2*π*/*N*.

In Figure 4, the tangential tension is minimal at the mass imperfection locations and maximal midway between adjacent mass imperfections. The maximum tangential tension decreases with increasing *N*. According to Equation (30), the total tangential tension is related to the radial displacement *υ_e_* and the rate of change in tangential displacement ∂*µ_e_*/∂*Θ*. The tangential tension *EAυ_e_*_0_/*R* of the perfect rotating resonant ring is a constant value determined by *υ_e_*_0_. The additional tangential tension caused by the mass imperfections is mainly related to ∂*µ_e_*/∂*Θ*. At the mass imperfection locations, although the radial displacement *υ_e_* reaches its maximum, *υ_e_*_0_ remains constant, and ∂*µ_e_*/∂*Θ* is minimal, resulting in both the additional tangential tension and the total tangential tension being minimized.

For the tangential tension *EAυ_e_*_0_/*R* of the perfect rotating ring, the radial displacement *υ_e_*_0_ in Equation (26) becomes extremely large as the rotational speed approaches $\sqrt{E/\rho R^{2}}$. The elastic force of the rotating ring is difficult to balance the centripetal acceleration, leading to unsteady deformation. We restrict the model’s rotational speed to below $\sqrt{E/\rho R^{2}}$. According to the basic parameters given in Table 1, this speed is approximately 50,000 rad/s.

**Figure 5:**
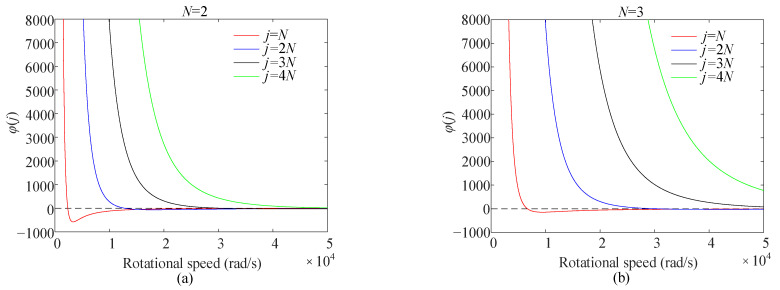
The variation in *φ*(*j*) with rotational speed for (**a**) *N* = 2 and (**b**) *N* = 3.

For the additional tangential tension caused by the mass imperfections, the tangential tension *N_Θ_* in Equation (26) becomes extremely large as *φ*(*j*) approaches zero. The rotational speed corresponding to *φ*(*j*) = 0 is the speed at which the deformation becomes unsteady. Figure 5 depicts the variation in *φ*(*j*) with respect to the rotational speed, considering only the cases below rotational speed $\sqrt{E/\rho R^{2}}$.

Figure 5 shows that the rotational speed corresponding to *φ*(*j*) = 0 becomes higher with an increasing *N*. For a given number of mass imperfections, the rotational speed corresponding to *φ*(*j*) = 0 is minimal. When *N* =8, the rotational speed corresponding to *φ*(*j*) = 0 exceeds $\sqrt{E/\rho R^{2}}$. The rotational speed at which the deformation becomes unsteady does not need to be considered during medium- and low-speed rotations. Increasing the number of the evenly distributed mass imperfections can enhance the stability of the steady elastic deformation of the ring resonator of the gyroscope.

### 3.3. Free Response

The inextensible assumption is utilized for analyzing low-speed rotating thin rings with lower-order modes for vibration gyros. Huang [9] compared the inextensional natural frequencies with those obtained without the inextensible assumption. At a rotational speed of 3600 rpm, the maximum difference between the two natural frequencies is less than 1.69%. However, this assumption is invalid for the problem of extremely high-order modes. Usually, the ring resonator of the gyroscope operates at lower-order modes with small vibration amplitudes, and thus, the ring resonator is assumed to be inextensional [25] and

(31) $$\frac{𝜕\mu }{𝜕\Theta }+\upsilon =0$$

Substituting Equations (14) and (15) into Equations (12) and (13) and eliminating the constant and nonlinear terms yield the system of partial differential equations of the vibration:

(32) $$\begin{array}{l} \left[\rho_{0}AR+m_{0}\sum_{p=1}^{N}\delta \left(\Theta -\Theta_{p}\right)\right]\left(\frac{{𝜕}^{2}\upsilon }{𝜕t^{2}}-2\frac{𝜕\mu }{𝜕t}\Omega -\Omega^{2}\upsilon \right)+\frac{EA}{R^{2}}\left(\mu_{e}-\frac{𝜕\upsilon_{e}}{𝜕\Theta }\right)\left(\mu -\frac{𝜕\upsilon }{𝜕\Theta }\right)\\ +\frac{EA}{R^{2}}\left[\left(\frac{{𝜕}^{2}\mu_{e}}{𝜕\Theta^{2}}+\frac{𝜕\upsilon_{e}}{𝜕\Theta }\right)\left(\mu -\frac{𝜕\upsilon }{𝜕\Theta }\right)+\left(\frac{{𝜕}^{2}\mu }{𝜕\Theta^{2}}+\frac{𝜕\upsilon }{𝜕\Theta }\right)\left(\mu_{e}-\frac{𝜕\upsilon_{e}}{𝜕\Theta }\right)\right]-\frac{EAh^{2}}{12R^{3}}\left(\frac{{𝜕}^{3}\mu }{𝜕\Theta^{3}}-\frac{{𝜕}^{4}\upsilon }{𝜕\Theta^{4}}\right)\\ +\frac{EA}{R^{2}}\left[\left(\frac{𝜕\mu_{e}}{𝜕\Theta }+\upsilon_{e}\left(\Theta \right)\right)\left(\frac{𝜕\mu }{𝜕\Theta }-\frac{{𝜕}^{2}\upsilon }{𝜕\Theta^{2}}\right)+\left(\frac{𝜕\mu }{𝜕\Theta }+\upsilon \right)\left(\frac{𝜕\mu_{e}}{𝜕\Theta }-\frac{{𝜕}^{2}\upsilon_{e}}{𝜕\Theta^{2}}\right)\right]+\frac{EA}{R}\left(\frac{𝜕\mu }{𝜕\Theta }+\upsilon \right)=0 \end{array}$$

(33) $$\begin{array}{l} \left[\rho_{0}AR+m_{0}\sum_{p=1}^{N}\delta \left(\Theta -\Theta_{p}\right)\right]\left(\frac{{𝜕}^{2}\mu }{𝜕t^{2}}+2\frac{𝜕\upsilon }{𝜕t}\Omega -\Omega^{2}\mu \right)-\frac{EA}{R}\left(\frac{{𝜕}^{2}\mu }{𝜕\Theta^{2}}+\frac{𝜕\upsilon }{𝜕\Theta }\right)\\ -\frac{EAh^{2}}{12R^{3}}\left(\frac{{𝜕}^{2}\mu }{𝜕\Theta^{2}}-\frac{{𝜕}^{3}\upsilon }{𝜕\Theta^{3}}\right)-\frac{EA}{R^{2}}\left[\left(\mu -\frac{𝜕\upsilon }{𝜕\Theta }\right)\left(\frac{𝜕\mu_{e}}{𝜕\Theta }-\frac{{𝜕}^{2}\upsilon_{e}}{𝜕\Theta^{2}}\right)+\left(\mu_{e}-\frac{𝜕\upsilon_{e}}{𝜕\Theta }\right)\left(\frac{𝜕\mu }{𝜕\Theta }-\frac{{𝜕}^{2}\upsilon }{𝜕\Theta^{2}}\right)\right]\\ +\frac{EA}{R^{2}}\left[\left(\frac{𝜕\mu }{𝜕\Theta }+\upsilon \right)\left(\mu_{e}-\frac{𝜕\upsilon_{e}}{𝜕\Theta }\right)+\left(\frac{𝜕\mu_{e}}{𝜕\Theta }+\upsilon_{e}\right)\left(\mu -\frac{𝜕\upsilon }{𝜕\Theta }\right)\right]=0 \end{array}$$

Substituting Equations (30) and (31) into Equations (32) and (33) gives the vibrational partial differential equation for the tangential displacement:

(34) $$\begin{array}{l} m_{0}\left(-\frac{{𝜕}^{3}\mu }{𝜕\Theta 𝜕t^{2}}-2\Omega \frac{𝜕\mu }{𝜕t}+\Omega^{2}\frac{𝜕\mu }{𝜕\Theta }\right)\sum_{p=1}^{N}\frac{𝜕\delta \left(\Theta -\Theta_{p}\right)}{𝜕\Theta }-\frac{EAh^{2}}{12R^{3}}\left(\frac{{𝜕}^{6}\mu }{𝜕\Theta^{6}}+2\frac{{𝜕}^{4}\mu }{𝜕\Theta^{4}}+\frac{{𝜕}^{2}\mu }{𝜕\Theta^{2}}\right)\\ +\left[\rho_{0}AR+m_{0}\sum_{p=1}^{N}\delta \left(\Theta -\Theta_{p}\right)\right]\left(-\frac{{𝜕}^{4}\mu }{𝜕\Theta^{2}𝜕t^{2}}+\Omega^{2}\frac{{𝜕}^{2}\mu }{𝜕\Theta^{2}}-4\Omega \frac{{𝜕}^{2}\mu }{𝜕\Theta 𝜕t}+\frac{{𝜕}^{2}\mu }{𝜕t^{2}}-\Omega^{2}\mu \right)\\ +\frac{1}{R}\left[\frac{{𝜕}^{2}N_{\Theta }}{𝜕\Theta^{2}}\left(\frac{{𝜕}^{2}\mu }{𝜕\Theta^{2}}+\mu \right)+2\frac{𝜕N_{\Theta }}{𝜕\Theta }\left(\frac{{𝜕}^{3}\mu }{𝜕\Theta^{3}}+\frac{𝜕\mu }{𝜕\Theta }\right)+N_{\Theta }\left(\frac{{𝜕}^{4}\mu }{𝜕\Theta^{4}}+2\frac{{𝜕}^{2}\mu }{𝜕\Theta^{2}}+\mu \right)\right]=0 \end{array}$$

By using Galerkin’s method, the tangential displacement *µ*(*Θ*, *t*) is written as

(35) $$\mu (\Theta ,t)=q_{n}\left(t\right)e^{in\Theta }+{\tilde{q}}_{n}\left(t\right)e^{-in\Theta }$$

where *q_n_*(*t*) is a complex function of the time *t*, *n* is the vibration wavenumber, *i* ($i=\sqrt{-1}$) is the imaginary unit, and “~” designates the complex conjugate operation.

To facilitate analysis, the complex function *q_n_*(*t*) is rewritten as

(36) $$q_{n}\left(t\right)=x\left(t\right)+iy\left(t\right)$$

where *x*(*t*) and *y*(*t*) are both real functions of the time.

An inner product is defined as

(37) $$<x,y>=\int_{0}^{2\pi }x\tilde{y}d\Theta$$

By substituting Equations (35) and (36) into Equation (34) and forming the inner product with *e^inΘ^*, the real and imaginary parts can be separated. Two cases are considered based on different relationships of *n* and *N*.

**Case Ι:** 2*n*/*N* = int

The governing equation in matrix differential operator form is

(38) $$M_{1}\ddot{\eta }\left(t\right)+G\dot{\eta }\left(t\right)+K_{1}\eta \left(t\right)=0$$

where

$$\begin{array}{c} \eta \left(t\right)=\left[\begin{array}{c} x\left(t\right)\\ y\left(t\right) \end{array}\right],\;M_{1}=\left[\begin{array}{cc} 1-\frac{K_{1}}{1+K_{1}}\frac{n^{2}-1}{n^{2}+1} & 0\\ 0 & 1+\frac{K_{1}}{1+K_{1}}\frac{n^{2}-1}{n^{2}+1} \end{array}\right],\;G=\left[\begin{array}{cc} 0 & \Omega \frac{4n}{n^{2}+1}\\ -\Omega \frac{4n}{n^{2}+1} & 0 \end{array}\right],\\ K_{1}=\left[\begin{array}{cc} -\Omega^{2}\left[f_{1}\left(n\right)+f_{2}\left(n\right)\right] & 0\\ 0 & -\Omega^{2}\left[f_{1}\left(n\right)+f_{2}\left(n\right)\right] \end{array}\right],\\ f_{1}\left(n\right)=1-\frac{K_{2}}{1+K_{1}}\frac{{\left(n^{2}-1\right)}^{2}}{n^{2}+1}+\frac{K_{2}h^{2}}{12\left(1+K_{1}\right)\left(1-K_{2}\right)R^{2}}\frac{n^{2}{\left(n^{2}-1\right)}^{2}}{n^{2}+1},\\ f_{2}\left(n\right)=\frac{K_{1}}{1+K_{1}}\frac{n^{2}-1}{n^{2}+1}\left[1+\frac{K_{2}^{2}\left(n^{2}-1\right)}{1-K_{2}}\frac{-b{\left(2n\right)}^{4}+b{\left(2n\right)}^{2}-1}{ab{\left(2n\right)}^{6}-\left(2ab+b\right){\left(2n\right)}^{4}-\left(a+b-ab\right){\left(2n\right)}^{2}+1-a}\right],\\ K_{1}=\frac{Nm_{0}}{2\pi \rho_{0}AR},\;K_{2}=\frac{E}{E-\rho_{0}R^{2}\Omega^{2}}. \end{array}$$

Equation (38) is the kinetic equation with constant coefficient, and the characteristics of the mode can be obtained through the eigenvalue analysis of the system. The state vector is

(39) $$\psi \left(t\right)=\left[\begin{array}{c} \eta \left(t\right)\\ \dot{\eta }\left(t\right) \end{array}\right]$$

Substituting Equation (39) into Equation (38) gives

(40) $$\dot{\psi }\left(t\right)=Q_{1}\psi \left(t\right)=\left[\begin{array}{cc} O & I\\ -M_{1}^{-1}K_{1} & -M_{1}^{-1}G \end{array}\right]\left[\begin{array}{c} \eta \left(t\right)\\ \dot{\eta }\left(t\right) \end{array}\right]$$

where $Q_{1}$ is the state space matrix and O and I represent the zero matrix and the identity matrix, respectively.

Assuming **ψ**(*t*) = *e^λt^***y**, where *λ* is the eigenvalue of the system and **y** is the eigenvector, the characteristic equation is

(41) $$\lambda y=\left[\begin{array}{cc} O & I\\ -M_{1}^{-1}K_{1} & -M_{1}^{-1}G \end{array}\right]y$$

According to the characteristic equation of the system, the equation for the eigenvalue *λ* can be expressed as

(42) $$\lambda^{4}+\Delta_{1}\lambda^{2}+\nabla_{1}=0$$

where

$$\begin{array}{c} \Delta_{1}=\frac{\Omega^{2}{\left(1+K_{1}\right)}^{2}{\left(4n\right)}^{2}}{\left(n^{2}+2K_{1}n^{2}+1\right)\left(n^{2}+2K_{1}+1\right)}-\left[f_{1}\left(n\right)+f_{2}\left(n\right)\right]\frac{2\Omega^{2}{\left(1+K_{1}\right)}^{2}{\left(n^{2}+1\right)}^{2}}{\left(n^{2}+2K_{1}n^{2}+1\right)\left(n^{2}+2K_{1}+1\right)},\\ \nabla_{1}=\Omega^{4}{\left[f_{1}\left(n\right)+f_{2}\left(n\right)\right]}^{2}\frac{{\left(1+K_{1}\right)}^{2}{\left(n^{2}+1\right)}^{2}}{\left(n^{2}+2K_{1}n^{2}+1\right)\left(n^{2}+2K_{1}+1\right)}. \end{array}$$

Thus, the eigenvalues are

(43) $$\lambda^{2}=\frac{-\Delta_{1}\pm \sqrt{\Delta_{1}^{2}-4\nabla_{1}}}{2}$$

Assuming *λ* = *λ*_Re_ + *iλ*_Im_, the real values *λ*_Re_ and *λ*_Im_ represent the real and imaginary parts of the eigenvalues, respectively. The eigenvalues for different conditions are summarized in Table 2.

**Case ΙΙ:** 2*n*/*N* ≠ int

The governing equation in matrix differential operator form is

(44) $$M_{2}\ddot{\eta }\left(t\right)+G\dot{\eta }\left(t\right)+K_{2}\eta \left(t\right)=0$$

where

$$M_{2}=\left[\begin{array}{cc} 1 & 0\\ 0 & 1 \end{array}\right],\;K_{2}=\left[\begin{array}{cc} -\Omega^{2}f_{1}\left(n\right) & 0\\ 0 & -\Omega^{2}f_{1}\left(n\right) \end{array}\right]$$

The corresponding characteristic equation is

(45) $$\lambda y=\left[\begin{array}{cc} O & I\\ -M_{2}^{-1}K_{2} & -M_{2}^{-1}G \end{array}\right]y$$

The equation for the eigenvalue *λ* is

(46) $$\lambda^{4}+\Delta_{2}\lambda^{2}+\nabla_{2}=0$$

where

$$\Delta_{2}=\Omega^{2}{\left(\frac{4n}{n^{2}+1}\right)}^{2}-2\Omega^{2}f_{1}\left(n\right),\;\nabla_{2}=\Omega^{4}f_{1}^{2}\left(n\right)$$

The eigenvalues can be obtained from Equation (46):

(47) $$\lambda^{2}=\frac{-\Delta_{2}\pm \sqrt{\Delta_{2}^{2}-4\nabla_{2}}}{2}$$

Substituting *λ* =*λ*_Re_ + *iλ*_Im_ into Equation (47), the eigenvalues are calculated and summarized in Table 3 for different conditions.

### 3.4. Qualitative Explanation of the Coriolis Effect

In fact, the literature has explained the Coriolis effect on the phenomenon that the forward and backward waves travel with different speeds on the rotating ring by the analytical and numerical results clearly, whereas a qualitative illustration is still added here, which may be useful for intuitive understanding.

Each point on the neutral plane moves along an elliptical trajectory when the ring rotates since the tangential and radial displacements are *u* = *A_n_*cos(*nθ* + *ω_n_t*) and *v* = *nA_n_*sin(*nθ* + *ω_n_t*), where *A_n_* is vibration amplitude and *ω_n_* is natural frequency. The tangential and radial displacements satisfy *u*^2^ + *v*^2^/*n*^2^ = 1, which corresponds to the ellipse.

**Figure 6:**
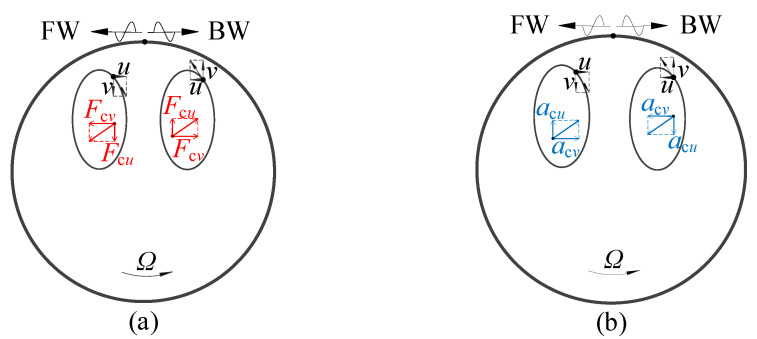
The Coriolis effect observed in (**a**) ground-fixed coordinates and (**b**) body-fixed coordinates.

The negative and positive natural frequencies correspond to the forward and backward waves, respectively. The clockwise motion of a point is present in Figure 6.

For the ground-fixed coordinates, the Coriolis effect should be analyzed based on the Coriolis force, which can be judged by the right-hand rule. In this figure, the Coriolis forces have the same direction as the displacement, and thus, the forward wave is accelerated just like the stiffness has increased, and the opposite is true for the backward wave. Therefore, the forward wave travels faster, as seen in the ground-fixed coordinates.

For the body-fixed coordinates, the Coriolis effect can be analyzed based on the Coriolis acceleration, which is in the opposite direction of the Coriolis force. Then, the result is the backward wave travels faster or the frequency of the backward wave is higher.

## 4. Numerical Results

### 4.1. Nature Frequencies and the Validation

**Figure 7:**
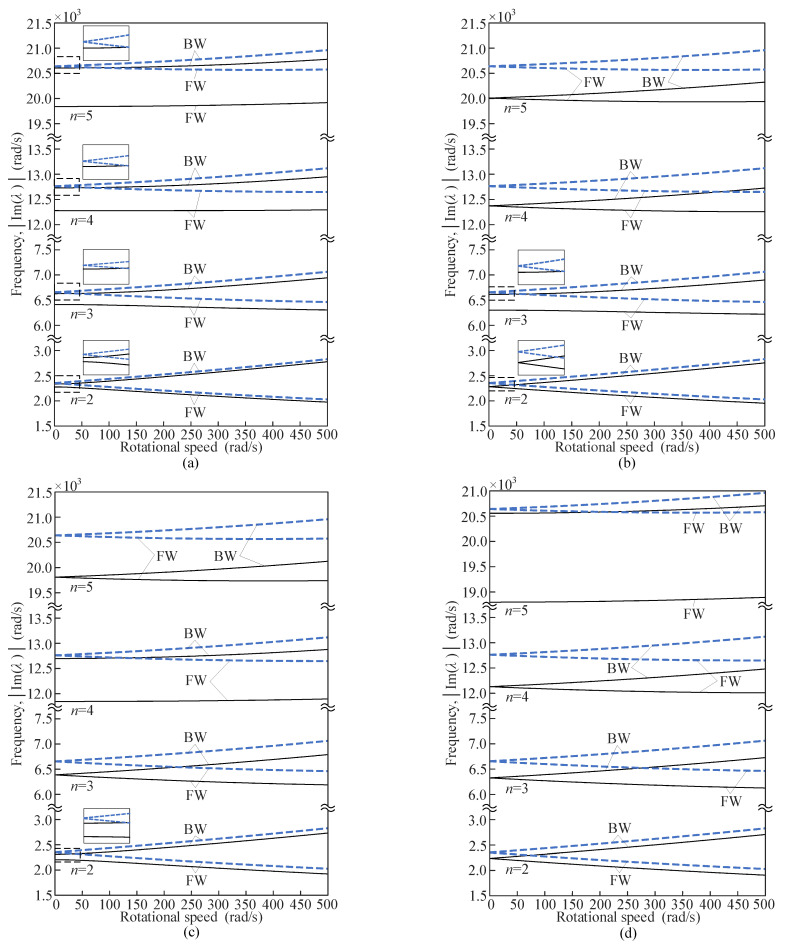
Natural frequencies of traveling waves in the ring-fixed coordinates, where the dashed curves ‘—’ denote the results for the perfect resonant ring and the straight curves ‘—’ denote the cases of (**a**) *N* = 2, (**b**) *N* = 3, (**c**) *N* = 4, and (**d**) *N* = 5.

The eigenvalues with parameters in Table 1 are computed numerically from the characteristic equation. The imaginary parts of the eigenvalues represent the natural frequencies of the free vibration in the ring-fixed coordinates. The natural frequencies for varying rotational speeds are shown in Figure 7, with the abscissa representing the rotational speeds and the ordinate representing the natural frequencies of forward traveling waves (FW) and backward traveling waves (BW).

As shown in Figure 7, the natural frequencies of the rotating ring resonator with evenly distributed mass imperfections are lower than those of the perfect rotating ring for the same wavenumber *n*. The natural frequencies will be reduced by the mass imperfections, with forward waves exhibiting a greater decrease than backward waves. In addition, the natural frequencies decrease with increasing *N* when the combinations of *n* and *N* belong to the same case (Ι or ΙΙ). Different relationships of *n* and *N* influence the natural frequency splitting at zero rotational speed. When 2*n*/*N* = int, the natural frequencies split. Specifically, the natural frequency of the BW of the rotating ring corresponds to the higher one of the split frequencies in the stationary state, and the natural frequency of the FW corresponds to the lower one. When 2*n*/*N* ≠ int, the natural frequencies do not split. The natural frequencies for a given wavenumber *n* of backward waves are greater than those of forward waves due to the Coriolis acceleration.

The finite element method (FEM) is applied to verify the numerical results by using the COMSOL Multiphysics 6.1 software, taking *N* = 4 as an illustrative example. The basic parameters in Table 1 are used for the simulation model. The physics interface selects the Solid Rotor (rotsld) in the Rotordynamics Module. This interface models the equations of motion for an observer sitting in a corotating frame of reference. Therefore, the natural frequencies solved by the finite element method are in the ring-fixed coordinates. The Coriolis and prestress effects are applied by setting the rotational speed. Table 4 gives the comparisons of natural frequencies (*N* = 4) obtained by the finite element method and numerical calculation.

The numerically calculated natural frequencies are circular frequencies *ω* (rad/s), and the frequencies in Table 4 are obtained by transforming *f* = *ω*/2*π* (Hz). Various rotational speeds are selected in the comparisons. For the case of zero rotational speed, the natural frequencies of the 3rd and 5th modes are equal to each other, but those of the 2nd and 4th modes are not due to the splitting of the natural frequencies (2*n*/*N* = int). When the ring resonator rotates, the natural frequency of each mode branches into two because of the Coriolis effect. It can be seen in Table 4 that the differences of the frequencies are small, and the maximal difference is less than 5%, which indicates the validity and accuracy of the model and approach.

The fully differential frequency-modulated gyroscopes measure the rotational speed based on the frequency differences between FW and BW by the Coriolis force. The frequencies of the two modes have the same temperature dependency, and the frequency fluctuation induced by temperature can be cancelled out for the frequency differences, resulting in excellent temperature stability [26,27,28]. The eigenfrequencies of the two modes are given as $f_{0}\pm k\Omega /2\pi$, where *f*_0_, *k*, and *Ω* are natural frequency in the stationary state, scale factor, and rotational speed, respectively.

**Figure 8:**
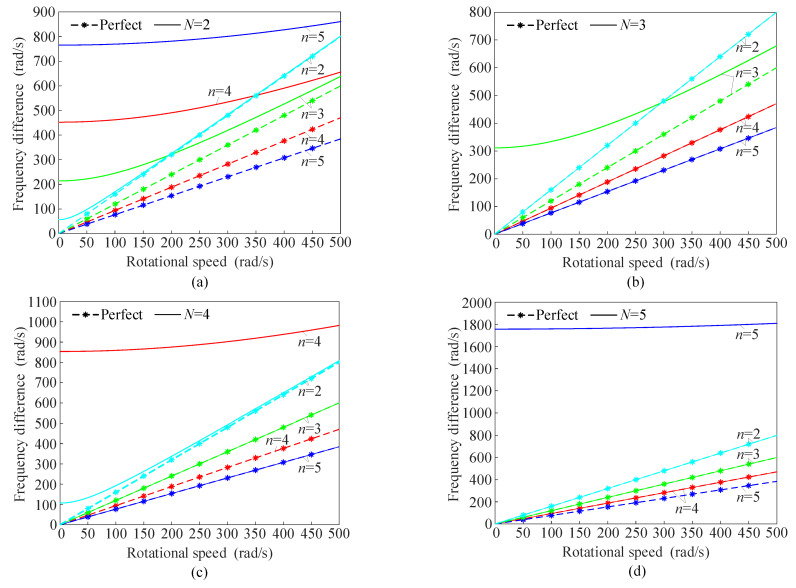
The frequency difference between FW and BW of the imperfect and perfect resonant rings for (**a**) *N* = 2, (**b**) *N* = 3, (**c**) *N* = 4, and (**d**) *N* = 5.

There is an excellent linearity between the applied rotational speeds and the frequency differences of the two modes for the perfect ring. Figure 8 shows the frequency differences between FW and BW based on the parameters in Table 1.

When 2*n*/*N* = int, the mass imperfections induce a significant discrepancy in the frequency differences between FW and BW of the imperfect and perfect resonant rings, especially in the low-speed range. This means that the mass imperfections introduce a non-negligible error in rotational speed measurements in case Ι (2*n*/*N* = int). For the perfect resonant rings, the frequency differences between BW and FW are caused by the Coriolis effect, while for the imperfect resonant rings, these differences are additionally affected by the frequency splitting. The frequency splitting results in larger frequency differences in the imperfect rings compared to the perfect ones. However, as the rotational speed increases, the frequency differences in the imperfect resonant rings gradually converge toward those of the perfect resonant rings. Furthermore, in the low-speed range, the mass imperfections also change the linearity between the frequency differences and the rotational speeds. When 2*n*/*N* ≠ int, the frequency differences between FW and BW are nearly identical in both the imperfect and perfect rings. For the ring resonator operating in the 2nd mode, the frequency-modulated gyroscope ensures high accuracy in rotational speed measurement when *N* > 4. Similarly, it is necessary to ensure that *N* > 6 for the 3rd mode. Increasing the number of the evenly distributed mass imperfections enhances the accuracy of the gyroscope for lower-order modes.

### 4.2. Crosspoints of the Natural Frequencies of FW and BW

**Figure 9:**
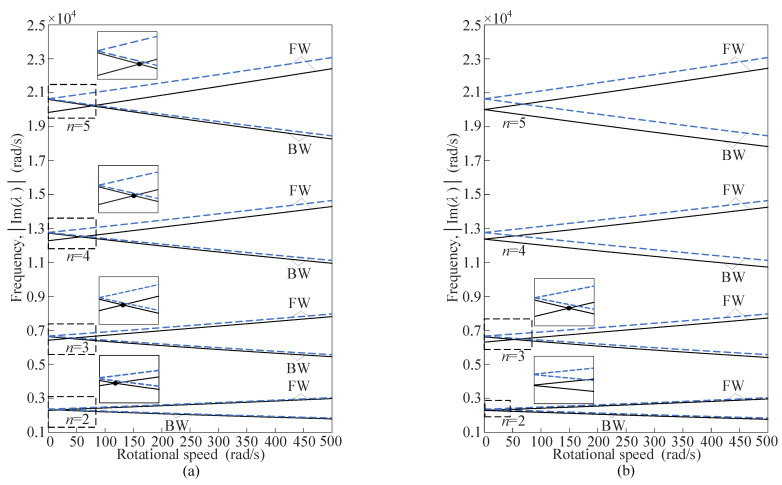

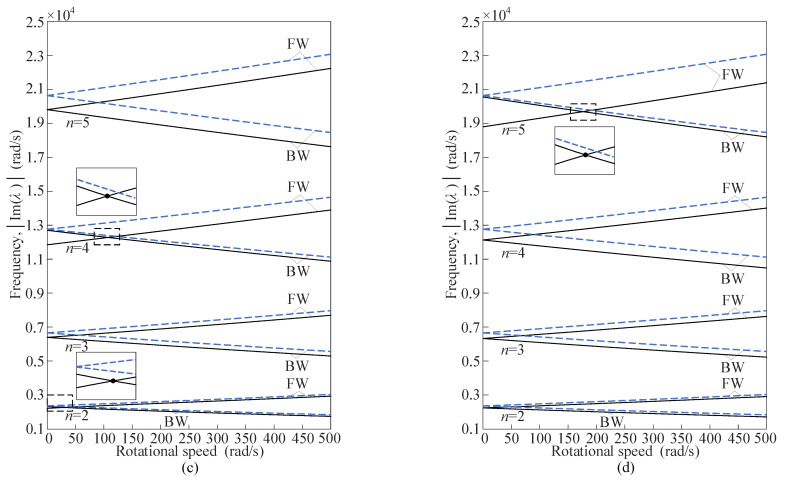
Natural frequencies of traveling waves in the ground-fixed coordinates, where the dashed curves ‘—’ denote the results for the perfect resonant ring and the straight curves ‘—’ denote the cases of (**a**) *N* = 2, (**b**) *N* = 3, (**c**) *N* = 4, and (**d**) *N* = 5. The dots ‘•’ denote the crosspoints of FW and BW for the imperfect resonant ring.

According to the Doppler effect, when converting the natural frequencies from the ring-fixed coordinates to the ground-fixed coordinates, the natural frequencies of forward waves increase by *nΩ*, whereas those of backward waves decrease by *nΩ*. Figure 9 shows the natural frequencies for varying rotational speeds in the ground-fixed coordinates.

In the ground-fixed coordinates, the natural frequencies of backward waves are always lower than those of forward waves for a given wavenumber *n* when 2*n*/*N* ≠ int. However, the situation of the natural frequencies is reversed between the high-speed and low-speed ranges when 2*n*/*N* = int. The frequency splitting causes the natural frequencies of backward waves to be higher than those of forward waves at zero rotational speed. In the low-speed range, the Doppler effect is not significant due to the low value of *nΩ*, and the natural frequencies of backward waves remain higher than those of forward waves because of the frequency splitting. As the rotational speed increases, the natural frequencies of forward waves gradually exceed those of backward waves. The transition in the magnitude relationship between the natural frequencies of forward and backward waves results in a crosspoint where their natural frequencies are equal to each other. For the perfect resonant rings, the crosspoints of the natural frequencies remain at zero rotational speed, without frequency splitting and the Coriolis effect.

**Figure 10:**
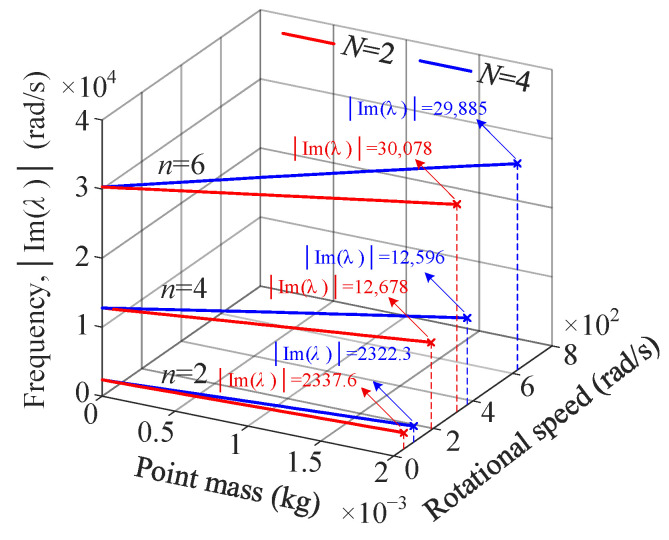
The effect of the mass imperfection on rotational speeds and natural frequencies at the crosspoints.

Figure 10 shows the variation in rotational speeds and natural frequencies at the crosspoints due to the increase in the magnitude of the mass imperfection.

Variation curves of wavenumbers *n* = 2, 4, and 6 are considered in Figure 10. Increasing the magnitude of the mass imperfection increases the rotational speeds at the crosspoints, while the natural frequencies exhibit an opposite trend. For the determined magnitude of the mass imperfection, larger values of *N* and *n* result in increased rotational speeds and natural frequencies at the crosspoints. When the magnitude of the mass imperfection approaches zero, the rotational speeds at the crosspoints also approach zero, since the natural frequencies of the perfect ring do not split in the stationary state.

## 5. Conclusions

This work derives a dynamic model for a rotating ring-shaped resonator in a gyroscope with evenly distributed mass imperfections, and the point masses are used to represent the mass imperfections. The effects of the evenly distributed mass imperfections on the steady elastic deformation and natural frequencies are investigated. The next step of this work aims to analyze the vibration of the model subjected to periodically fluctuated rotational speed as well as the nonlinear property considering large vibration amplitude. Below, the main conclusions of this work are summarized:

(1) The angular period of the displacements and tangential tension for the steady elastic deformation is 2*π*/*N*, and both the amplitudes of the displacements and tangential tension decrease with increasing *N*. There are multiple rotational speeds at which the deformation is unstable due to the mass imperfections, while the perfect rotating ring has only one. Increasing the number of evenly distributed mass imperfections can enhance the stability of the steady elastic deformation of the rotating imperfect ring.

(2) The natural frequencies of the stationary resonant ring split for integer 2*n*/*N* or remain degenerate for non-integer. The frequency splitting results in crosspoints of the natural frequencies of the FW and BW, and the magnitude of the mass imperfection can influence the rotational speeds and the natural frequencies at crosspoints. When 2*n*/*N* =int, the natural frequency of the BW of the rotating ring begins with the higher one of the split frequencies in the stationary state, and the natural frequency of the FW begins with the lower one.

(3) The mass imperfections reduce the natural frequencies of the rotating resonant ring in a gyroscope. When 2*n*/*N* = int, the mass imperfections will induce a significant discrepancy in the frequency differences between FW and BW of the imperfect and perfect rings, bringing a non-negligible error to the frequency-modulated gyroscope. When 2*n*/*N* ≠ int, the frequency differences between FW and BW are nearly identical in both the imperfect and perfect rings. The comparison of natural frequencies obtained from the finite element method and numerical calculation reveals a maximum discrepancy of less than 5%.

## Figures and Tables

**Table 1:** The parameters of the model.

Parameters	Values and Units
Neutral circle radius *R*	0.1 m
Axial length *b*	0.01 m
Young’s modulus *E*	2.0 × 10^11^ N/m^2^
Density *ρ*	7.8 × 10^3^ kg/m^3^
Thickness *h*	6 × 10^−3^ m
Magnitude of mass imperfection *m*_0_	2π × 10^−3^ kg
Rotational speed *Ω*	500 rad/s

**Table 2:** The eigenvalues of the vibration of the system at 2*n*/*N* = int.

Conditions		*λ*_Re_	*λ*_Im_
$\Delta_{1}^{2}-4\nabla_{1}>0$	$-\Delta_{1}\pm \sqrt{\Delta_{1}^{2}-4\nabla_{1}}>0$	$\pm \frac{\sqrt{2}}{2}\sqrt{-\Delta_{1}\pm \sqrt{\Delta_{1}^{2}-4\nabla_{1}}}$	0
	$-\Delta_{1}\pm \sqrt{\Delta_{1}^{2}-4\nabla_{1}}<0$	0	$\pm \frac{\sqrt{2}}{2}\sqrt{\Delta_{1}\mp \sqrt{\Delta_{1}^{2}-4\nabla_{1}}}$
$\Delta_{1}^{2}-4\nabla_{1}<0$	$\Delta_{1}-2\sqrt{\nabla_{1}}<0$	$\pm \frac{1}{2}\sqrt{2\sqrt{\nabla_{1}}-\Delta_{1}}$	$\pm \frac{1}{2}\frac{\sqrt{-\left(\Delta_{1}^{2}-4\nabla_{1}\right)}}{\sqrt{2\sqrt{\nabla_{1}}-\Delta_{1}}}$
	$\Delta_{1}+2\sqrt{\nabla_{1}}>0$	$\pm \frac{1}{2}\frac{\sqrt{-\left(\Delta_{1}^{2}-4\nabla_{1}\right)}}{\sqrt{2\sqrt{\nabla_{1}}+\Delta_{1}}}$	$\pm \frac{1}{2}\sqrt{2\sqrt{\nabla_{1}}+\Delta_{1}}$

**Table 3:** The eigenvalues of the vibration of the system at 2*n*/*N* ≠ int.

Conditions		*λ*_Re_	*λ*_Im_
$\Delta_{2}^{2}-4\nabla_{2}>0$	$-\Delta_{2}\pm \sqrt{\Delta_{2}^{2}-4\nabla_{2}}>0$	$\pm \frac{\sqrt{2}}{2}\sqrt{-\Delta_{2}\pm \sqrt{\Delta_{2}^{2}-4\nabla_{2}}}$	0
	$-\Delta_{2}\pm \sqrt{\Delta_{2}^{2}-4\nabla_{2}}<0$	0	$\pm \frac{\sqrt{2}}{2}\sqrt{\Delta_{2}\mp \sqrt{\Delta_{2}^{2}-4\nabla_{2}}}$
$\Delta_{2}^{2}-4\nabla_{2}<0$	$\Delta_{2}-2\sqrt{\nabla_{2}}<0$	$\pm \frac{1}{2}\sqrt{2\sqrt{\nabla_{2}}-\Delta_{2}}$	$\pm \frac{1}{2}\frac{\sqrt{-\left(\Delta_{2}^{2}-4\nabla_{2}\right)}}{\sqrt{2\sqrt{\nabla_{2}}-\Delta_{2}}}$
	$\Delta_{2}+2\sqrt{\nabla_{2}}>0$	$\pm \frac{1}{2}\frac{\sqrt{-\left(\Delta_{2}^{2}-4\nabla_{2}\right)}}{\sqrt{2\sqrt{\nabla_{2}}+\Delta_{2}}}$	$\pm \frac{1}{2}\sqrt{2\sqrt{\nabla_{2}}+\Delta_{2}}$

**Table 4:** Comparisons of natural frequencies (*N* = 4) between the finite element method and numerical calculation.

Modes *n*	*Ω* (rad/s)	*f*_FW_ (Hz)			*f*_BW_ (Hz)		
		FEM	Numerical	Diff. (%)	FEM	Numerical	Diff. (%)
2	0	350.74	351.30	0.16	367.72	368.32	0.16
	250	325.50	329.55	1.22	391.45	395.52	1.03
	500	291.56	306.28	4.81	421.81	434.91	3.01
3	0	1012.70	1016.83	0.41	1012.70	1016.83	0.41
	250	987.99	997.04	0.91	1035.80	1044.79	0.86
	500	966.23	985.33	1.94	1061.10	1080.82	1.82
4	0	1868.30	1885.35	0.90	2004.70	2021.11	0.81
	250	1865.50	1887.26	1.15	2006.50	2028.43	1.08
	500	1860.00	1893.47	1.77	2024.90	2049.76	1.21
5	0	3121.80	3153.02	0.99	3121.80	3153.02	0.99
	250	3104.00	3142.67	1.23	3135.00	3173.23	1.20
	500	3103.20	3142.04	1.23	3148.40	3203.31	1.71

Notes: Diff. = (Numerical-FEM)/Numerical × 100%, *f*_FW_ and *f*_BW_ represent the natural frequencies for the forward and backward waves, respectively.

## Data Availability

The detailed information supporting the report’s findings can be found in Table 4 of the paper.
